# Systemic immune-inflammation index as a predictor of all-cause mortality in patients with hepatitis B virus infection: A cross-sectional study based on NHANES 1999 to 2018

**DOI:** 10.1097/MD.0000000000046305

**Published:** 2026-05-12

**Authors:** Min Chen, Yu Tang, Jiameng Li, Li Qiang, Gang Wu

**Affiliations:** a Department of Infectious Disease, The Affiliated Hospital of Southwest Medical University, Jiangyang District, Luzhou, Sichuan, China.

**Keywords:** cross-sectional study, hepatitis B virus infection, NHANES, prediction, systemic immune-inflammation index

## Abstract

Although the Systemic Immune-Inflammation Index (SII) has established prognostic value in several diseases, its role in hepatitis B virus (HBV) infection remains undefined. HBV-related inflammation drives progression to cirrhosis, hepatic failure, and hepatocellular carcinoma. However, the association between SII and all-cause mortality in this population has not been well established. Therefore, our study aims to investigate the association of SII with all-cause mortality in patients with HBV infection. We retrospectively analyzed a cohort of US adults (≥18 years) with HBV infection from the 1999 to 2018 National Health and Nutrition Examination Survey, focusing specifically on HBeAg-positive individuals. All-cause mortality (ascertained through the National Death Index as of December 2018) was evaluated in relation to SII using multivariable Cox regression with restricted cubic splines. Prognostic accuracy was evaluated using time-dependent receiver operating characteristic analysis, complemented by Kaplan–Meier survival estimates. Subgroup and sensitivity analyses verified the robustness of the results against potential confounders. The cohort comprised 3332 HBV-infected individuals followed for a mean 105.83 ± 65.21 months, during which 682 all-cause mortality events were recorded. In the fully adjusted Cox model, a higher Log-Transformed Systemic Immune-Inflammation Index (lnSII) level remained independently associated with a significant 53% increase in all-cause mortality (hazard ratio = 1.53, 95% confidence interval: 1.13–2.08). Restricted cubic spline regression confirmed a dose-dependent positive association between lnSII and mortality (*P* < .001). Time-dependent receiver operating characteristic analysis demonstrated sustained high discriminatory accuracy across all intervals (area under the curve: 0.895 at 1-year, 0.874 at 3-year, 0.870 at 5-year, 0.868 at 10-year). Subgroup analyses revealed pronounced risk amplification among Hispanic individuals (hazard ratio = 2.90, 95% confidence interval: 1.14–7.38), while a U-shaped mortality relationship manifested in the low hepatic fibrosis stratum (Fibrosis-4 index < 1.30; *P* = .006), with optimal survival at lnSII = 6. Robustness was confirmed through extensive sensitivity analyses. Elevated SII is an independent predictor of all-cause mortality in HBV-infected patients, with the greatest risk in the top quartile. Its consistent accuracy for both short- and long-term outcomes calls for integrating this marker into clinical practice to improve risk-stratified management.

## 1. Introduction

Hepatitis B virus (HBV), a hepatotropic and non-cytopathogenic DNA virus, represents the primary etiological agent of viral hepatitis globally.^[1]^ Globally, approximately 296 million people live with chronic HBV infection. Although epidemiological projections suggest that the number of infections will decline to around 267 million by 2030, HBV-related mortality is expected to continue rising, reaching approximately 1.11 million deaths per year.^[2,3]^ This infection may progress to acute or chronic hepatitis, severe hepatic failure, or fatal outcomes.^[4]^ Global vaccination efforts have markedly decreased hepatitis B incidence and prevalence, evidenced by a 31.3% reduction in chronic infection rates (1990–2019) and 68 countries achieving the WHO’s 2030 mortality reduction goal.^[5]^ Nevertheless, HBV prevention and control efforts in resource-limited regions face persistent obstacles. These challenges include low vaccination coverage, inadequate diagnostic capacity, limited access to affordable treatment, and weak health system infrastructure.^[6–8]^ The Model for End Stage Liver Disease and Child Turcotte Pugh scores, currently the most widely adopted clinical tools, effectively quantify hepatic functional impairment but fail to capture systemic inflammatory states and immune dysregulation. Critically, mounting evidence demonstrates that systemic inflammation drives HBV chronicity, accelerates liver fibrosis progression, and triggers acute exacerbations.^[9,10]^ Consequently, the identification of novel, cost-effective, and readily accessible prognostic biomarkers represents a pressing clinical imperative to address the global burden of HBV disease.

The Systemic Immune-Inflammation Index (SII) is a composite biomarker that integrates absolute neutrophil, lymphocyte, and platelet counts, providing a quantitative measure of systemic inflammatory status and immune dysregulation.^[11]^ In the pathophysiology of HBV infection, these cellular components exhibit divergent pathophysiological roles. Elevated neutrophil and platelet counts reflect a sustained pro-inflammatory state that perpetuates hepatic injury and drives disease progression.^[12–15]^ Research demonstrates that peripheral lymphocyte enumeration and functional competence in HBV-infected individuals correlate strongly with disease progression.^[16]^ Effector T cells directly target and eliminate infected hepatocytes, while CD4⁺ T-helper cells orchestrate immune responses through cytokine secretion.^[17,18]^ Thus, SII provides a novel composite biomarker delineating dysregulation between pro-inflammatory states and antiviral immunity.

SII has been validated as a robust prognostic indicator across multiple malignancies and cardiovascular pathologies, demonstrating significant utility in predicting disease progression and clinical outcomes.^[19,20]^ While an elevated SII is well established as a predictor of short-term outcomes in HBV-related acute-on-chronic liver failure, existing studies have been limited to follow-up periods of 28 or 90 days.^[21,22]^ It remains unknown whether SII also predicts long-term risk in the broader chronic HBV population: particularly all-cause mortality. To examine this, we analyzed a nationally representative cohort of U.S. adults with chronic HBV infection over a median follow-up of 8.8 years. This extended timeframe allows for the assessment of all-cause mortality, a definitive endpoint that reflects the cumulative burden of HBV-related sequelae such as cirrhosis and hepatocellular carcinoma (HCC), which develop over years.^[23]^ Identifying patients at elevated long-term mortality risk is crucial for personalizing surveillance and management. Our study was specifically designed to evaluate the association between SII and all-cause mortality in order to address this unresolved question.

## 2. Materials and methods

### 2.1. Data sources and study population

**Figure 1.**
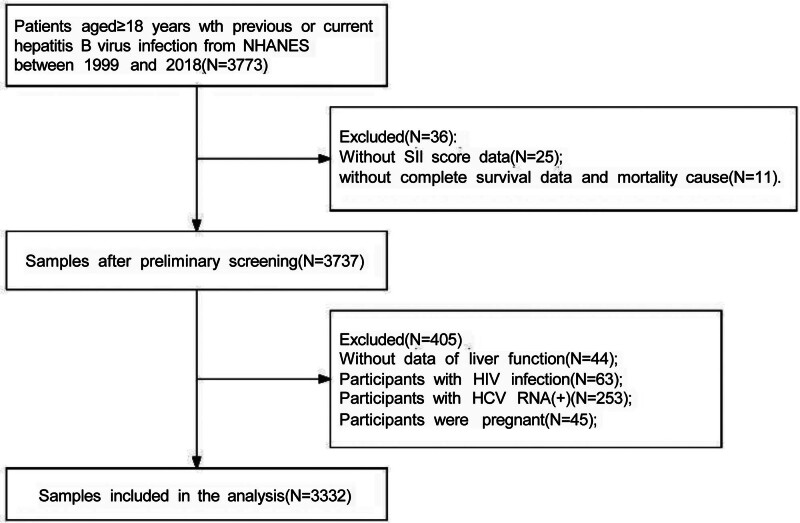
Participant enrollment flowchart for the study of SII and mortality in HBV infection. Anti-HBc = antibody to hepatitis B core antigen; HBV = hepatitis B virus; HBsAg = hepatitis B surface antigen; HCV = hepatitis C virus; HIV = human immunodeficiency virus; SII = Systemic Immune-Inflammation Index.

The National Health and Nutrition Examination Survey (NHANES) is a cross-sectional surveillance program assessing health and nutritional parameters in the non-institutionalized U.S. population. Data collection incorporates structured interviews, standardized physical examinations, dietary assessments, and comprehensive laboratory testing. Publicly accessible data are available through the official repository (https://www.cdc.gov/nchs/nhanes/index.htm). We analyzed data from ten biennial cycles (1999–2018) of the NHANES. This survey employs a stratified, multistage probability sampling design to recruit a nationally representative sample of the non-institutionalized U.S. population, with each cycle including approximately 5000 examined participants. The initial pooled sample across cycles comprised 101,316 individuals. The breadth of NHANES data (encompassing hepatitis B serology, complete blood count parameters, linked mortality records, and a wide array of covariates) renders it particularly appropriate for fulfilling the aims of this study. The analysis included adults (≥18 years) with serological evidence of current or past hepatitis B virus infection, defined as positivity for hepatitis B surface antigen (HBsAg) or anti-HBc. Exclusion criteria comprised missing data necessary for calculating the SII or determining survival status, as well as the presence of missing liver function tests, concurrent human immunodeficiency virus infection, detectable hepatitis C virus RNA, or pregnancy. The final analytical cohort consisted of 3332 eligible participants, with the complete selection process detailed in Figure 1. The dataset supporting the findings of this study is available as Data S1, Supplemental Digital Content, https://links.lww.com/MD/Q824. As this research utilized a publicly available de-identified database, the National Center for Health Statistics Research Ethics Review Board granted a formal waiver for ethical review.

### 2.2. SII and outcome definitions

Complete blood counts were performed using a Coulter DxH 800 analyzer operated by certified laboratory personnel. Based on these results, the SII was calculated as: SII = [platelet count (×10⁹/L) × neutrophil count (×10⁹/L)]/lymphocyte count (×10⁹/L). The primary study endpoint (all-cause mortality) was ascertained through linkage to the National Death Index (https://www.cdc.gov/nchs/data-linkage/mortality-public.htm), with follow-up through December 2019. With a sensitivity of 98.4% in the general U.S. population, the National Death Index provides a comprehensive and reliable database for mortality ascertainment.^[24]^ The selected follow-up period provided a minimum potential observation time of approximately 1 year for all participants, even those enrolled in the latest (2018) survey cycle, thus allowing for the ascertainment of mortality. For cause-of-death classification, International Classification of Diseases, 10th Revision codes were used for all mortality records.

### 2.3. Covariate assessment

Demographic characteristics (including age, sex (male/female), race/ethnicity (Mexican American, non-Hispanic White, non-Hispanic Black, other Hispanic, other race), educational attainment (>high school, ≤high school), and poverty-to-income ratio (≤1.0, 1.0–3.0, >3.0)) were ascertained through structured NHANES questionnaires. Complementing these self-reported data, standardized anthropometric measurements (standing height, body weight, waist circumference) were obtained at Mobile Examination Centers by trained personnel using calibrated instruments. Subsequently, body mass index (BMI; kg/m²) was calculated as weight/height² and categorized per WHO criteria into underweight (<18.5), normal weight (18.5–24.9), overweight (25.0–29.9), and obesity (≥30.0). Risk factor assessment captured physician-diagnosed hypertension, diabetes mellitus, cardiovascular disease (CVD) history, smoking status, and alcohol consumption via health questionnaires. Specifically, smoking status stratified participants into never (<100 lifetime cigarettes), former (*≥*100 lifetime cigarettes but reporting no current smoking at the time of survey), and current smokers (≥100 cigarettes with nondaily/daily use); alcohol use was dichotomized into “no use” (zero intake past year) versus “any use.” To standardize disease definitions, hypertension required self-reported diagnosis, antihypertensive medication, or measured BP ≥ 140/90 mm Hg; diabetes mellitus was defined by professional diagnosis, hypoglycemic medication, fasting glucose > 126 mg/dL, or HbA1c ≥ 6.5%; CVD history mandated physician-documented coronary heart disease, angina, or myocardial infarction. Laboratory analyses quantified hematological indices (neutrophil/lymphocyte/monocyte/platelet counts), hepatic biomarkers (ALT, AST, GGT, ALP, albumin, total bilirubin), metabolic measures (sodium, creatinine), and HBsAg serostatus from blood specimens under standardized protocols. Additionally, the Fibrosis-4 (FIB-4) index, a noninvasive marker of liver fibrosis, was calculated for each participant using the established formula: FIB-4 = [AST (IU/L) × age (years)]/[platelet count (10⁹/L) × √ALT (IU/L)].^[25]^

### 2.4. Statistical analysis

All statistical analyses were performed using R version 4.5 (R Foundation for Statistical Computing, Vienna, Austria). In accordance with NHANES analytical guidelines, we incorporated survey weights, stratification, and clustering variables to account for the complex, multistage probability sampling design and to generate nationally representative estimates. For variable presentation, continuous variables conforming to normal distribution are reported as mean ± standard deviation, and categorical variables as percentages with 95% confidence interval (CIs). Given the right-skewed distribution of SII, we performed log₂-transformation before categorizing it into quartile-based subgroups for subsequent analyses. Inferential statistics utilized independent samples t-tests for normally distributed continuous variables, whereas Mann–Whitney *U* or Kruskal–Wallis tests were applied to non-normally distributed variables based on group characteristics.

Potential predictors of all-cause mortality were 1st identified through weighted univariable Cox proportional hazards regression. Variables attaining statistical significance were retained for multivariable analysis using weighted Cox regression to evaluate the Log-Transformed Systemic Immune-Inflammation Index (lnSII)–mortality relationship. This analysis employed sequentially adjusted models: a minimally adjusted crude model; Model 1 (sociodemographic factors: age, sex, education, race/ethnicity, income); Model 2 (additionally adjusted for smoking, AST, GGT, ALP, albumin, creatinine, FIB-4); and Model 3 (further adjusted for BMI, diabetes, hypertension, CVD, and HBsAg status).

To investigate the dose–response relationship between SII and mortality, multivariable-adjusted restricted cubic splines (RCS) regression was employed. Concurrently, Kaplan–Meier curves, stratified by SII quartiles, were used to assess survival probability distributions with the log-rank test, while the predictive accuracy of lnSII was quantified using the “timeROC” R package.

Subgroup analyses were stratified by sex, race/ethnicity, educational attainment, household income, alcohol use status, smoking status, diabetes, hypertension, CVD, and BMI. Concurrently, we assessed potential interactions between these stratification factors and lnSII. Given the established role of liver fibrosis severity as a strong prognostic factor in HBV infection, RCS regression was additionally performed with FIB-4 stratification.

Robustness of the findings was evaluated through 3 sequential sensitivity analyses. First, participants who died within the initial 2-year follow-up period were excluded to mitigate potential reverse causation. Second, to mitigate selection bias and preserve statistical power, we implemented multiple imputation for missing data. Ten datasets were created via chained equations, incorporating all study variables. This approach reduces bias under the missing-at-random assumption while maintaining sample size. We subsequently reanalyzed the SII–mortality relationship by pooling estimates across all imputed datasets to obtain more robust effect estimates. Third, *E*-values quantified the potential impact of unmeasured confounding, representing the minimum strength of association needed to fully explain the observed exposure–outcome relationship.^[26]^ Statistical significance was defined as 2-sided *P* < .05.

## 3. Results

### 3.1. Baseline participant characteristics

From 1999 to 2018, this study enrolled 3332 adults with current or chronic HBV infection, with a mean age of 56.9 ± 16.0 years. Males constituted 55.6% (95% CI: 53.9–57.3) of the cohort, and females constituted 44.4% (95% CI: 42.7–46.1). Weighted baseline demographic characteristics of the study population are presented in Table 1. The weighted mean lnSII was 6.06 (95% CI: 6.03–6.08), corresponding to an SII value of 428. Participants with elevated SII were predominantly non-Hispanic Black. Compared to the low-SII group, this cohort demonstrated a significantly higher prevalence of hypertension and cardiovascular disease, elevated levels of neutrophil, monocyte, and platelet counts, advanced hepatic fibrosis as assessed by the FIB-4 index, and increased HBsAg seropositivity. In contrast, no significant differences were observed for age, GGT, ALP, albumin, sodium, follow-up time, gender, education level, poverty-to-income ratio, alcohol consumption, smoking status, BMI, diabetes, or hyperlipidemia across SII quartiles (all *P* > .05).

**Table 1:** Baseline characteristics of US adults with hepatitis B virus infection (NHANES 1999–2018) by quartiles of lnSII.

Variables	Q1 (<5.7)	Q2 (5.7–6.0)	Q3 (6.0–6.4)	Q4 (>6.4)	*P*-value
Age (yr)	52.40 ± 15.29	53.57 ± 15.94	53.65 ± 15.40	54.31 ± 16.50	.234
Gender (%)					.213
Male	58.0% (53.4–62.5)	55.3% (50.6–60.0)	50.9% (46.3–55.6)	54.3% (49.8–58.9)	
Female	42.0% (37.5–46.6)	44.7% (40.0–49.4)	49.1% (44.4–53.7)	45.7% (41.1–50.2)	
Ethnicity (%)					<.001
Mexican American	3.0% (2.0–4.1)	3.8% (2.6–5.0)	3.0% (2.1–3.9)	4.2% (3.0–5.3)	
Other Hispanic	6.2% (4.3–8.1)	8.7% (6.0–11.3)	8.4% (6.4–10.4)	6.7% (4.6–8.7)	
Non-Hispanic White	36.7% (32.7–40.7)	24.4% (21.2–27.6)	19.3% (16.6–21.9)	19.4% (16.6–22.1)	
Non-Hispanic Black	28.6% (23.1–34.0)	35.4% (30.2–40.7)	38.6% (33.7–43.5)	47.4% (42.8–52.0)	
Other	25.5% (21.7–29.2)	27.7% (23.8–31.6)	30.8% (26.5–35.0)	22.4% (18.8–26.1)	
Education level (%)					.769
<High school	26.8% (23.1–30.5)	29.2% (25.2–33.1)	29.5% (25.6–33.4)	29.0% (25.2–32.9)	
>High school	73.2% (69.5–76.9)	70.8% (66.9–74.8)	70.5% (66.6–74.4)	71.0% (67.1–74.8)	
PIR (%)					.216
Low	23.2% (19.6–26.8)	20.0% (16.8–23.2)	22.7% (18.9–26.4)	20.6% (17.3–23.9)	
Middle	43.7% (39.1–48.4)	40.4% (35.8–45.0)	40.0% (35.6–44.4)	46.2% (41.7–50.7)	
High	33.1% (28.3–37.9)	39.6% (34.6–44.6)	37.4% (32.7–42.0)	33.2% (28.7–37.8)	
Smoking (%)					.069
Current	20.4% (16.6–24.2)	24.2% (19.9–28.5)	23.5% (19.3–27.7)	28.5% (24.2–32.7)	
Former	26.5% (22.0–31.0)	23.9% (20.0–27.7)	22.7% (19.0–26.3)	26.1% (22.2–30.1)	
Never	53.2% (48.4–57.9)	52.0% (47.2–56.7)	53.9% (49.3–58.5)	45.4% (40.9–49.9)	
Drink (%)					.639
No	34.0% (29.7–38.3)	31.9% (27.4–36.4)	35.3% (30.9–39.7)	32.1% (28.0–36.2)	
Yes	66.0% (61.7–70.3)	68.1% (63.6–72.6)	64.7% (60.3–69.1)	67.9% (63.8–72.0)	
BMI (kg/m^2^, %)					.379
Underweight	3.4% (1.4–5.3)	1.6% (0.6–2.5)	2.3% (0.9–3.8)	2.5% (0.9–4.1)	
Normal	36.4% (31.9–41.0)	32.9% (28.6–37.3)	34.6% (30.2–39.1)	37.2% (32.9–41.6)	
Overweight	36.3% (31.6–41.0)	35.9% (31.2–40.5)	33.1% (28.8–37.4)	31.4% (27.2–35.6)	
Obese	23.9% (20.2–27.7)	29.6% (25.2–34.1)	30.0% (25.8–34.2)	28.9% (24.6–33.1)	
Diabetes (%)					.589
No	82.9% (79.6–86.1)	83.9% (80.9–86.9)	82.1% (79.0–85.2)	80.8% (77.5–84.2)	
Yes	17.1% (13.9–20.4)	16.1% (13.1–19.1)	17.9% (14.8–21.0)	19.2% (15.8–22.5)	
Hyperlipidemia (%)					.054
No	59.7% (55.3–64.1)	57.1% (52.4–61.9)	56.7% (52.2–61.2)	50.9% (46.4–55.5)	
Yes	40.3% (35.9–44.7)	42.9% (38.1–47.6)	43.3% (38.8–47.8)	49.1% (44.5–53.6)	
Cardiovascular (%)					<.001
No	94.9% (93.0–96.9)	93.5% (91.5–95.6)	93.8% (92.0–95.6)	88.2% (85.3–91.1)	
Yes	5.1% (3.1–7.0)	6.5% (4.4–8.5)	6.2% (4.4–8.0)	11.8% (8.9–14.7)	
Follow time (mo)	110.58 ± 66.80	115.54 ± 69.07	121.95 ± 66.88	118.31 ± 70.35	.308
HBsAg (%)					.002
Negative	88.9% (86.2–91.7)	93.8% (92.0–95.7)	94.6% (92.6–96.6)	94.0% (91.9–96.0)	
Positive	11.1% (8.3–13.8)	6.2% (4.3–8.0)	5.4% (3.4–7.4)	6.0% (4.0–8.1)	
Mortstat (%)					<.001
Alive	86.1% (83.1–89.1)	87.0% (84.2–89.8)	81.3% (78.1–84.6)	75.1% (71.4–78.9)	
Dead	13.9% (10.9–16.9)	13.0% (10.2–15.8)	18.7% (15.4–21.9)	24.9% (21.1–28.6)	
NEU (10⁹/L)	2.57 ± 0.84	3.47 ± 0.96	4.06 ± 1.05	5.53 ± 1.68	<.001
LYM (10⁹/L)	2.40 ± 0.99	2.27 ± 0.69	2.12 ± 0.65	1.85 ± 0.62	.027
MO (10⁹/L)	0.49 ± 0.23	0.55 ± 0.19	0.54 ± 0.17	0.60 ± 0.21	<.001
PLT (10⁹/L)	197.93 ± 52.64	231.67 ± 45.66	257.06 ± 55.23	287.85 ± 70.84	<.001
ALT (U/L)	29.65 ± 27.60	25.40 ± 20.25	24.63 ± 15.51	24.55 ± 47.61	.025
AST (U/L)	30.56 ± 31.72	25.79 ± 16.57	24.47 ± 9.87	25.22 ± 40.08	.003
GGT (IU/L)	35.56 ± 47.00	32.97 ± 46.68	30.49 ± 42.62	30.59 ± 33.20	.433
ALP (IU/L)	69.64 ± 27.82	69.33 ± 21.50	72.18 ± 27.20	73.54 ± 26.72	.866
Albumin (g/L)	42.61 ± 3.69	42.60 ± 3.26	42.49 ± 3.30	42.30 ± 3.60	.959
TBil (μmol/L)	12.21 ± 5.24	11.04 ± 4.67	11.41 ± 4.80	11.28 ± 5.12	<.001
Scr (μmol/L)	82.52 ± 43.75	77.57 ± 49.31	77.79 ± 32.21	81.79 ± 38.48	.031
Na (mmol/L)	139.70 ± 2.23	139.45 ± 2.55	139.42 ± 2.36	139.30 ± 2.47	.096
FIB-4	1.77 ± 1.78	1.28 ± 0.68	1.14 ± 0.57	1.07 ± 0.63	<.001

ALP = alkaline phosphatase, ALT = alanine aminotransferase, AST = aspartate aminotransferase, FIB-4 = Fibrosis-4 Index, GGT = gamma-glutamyl transferase, HBsAg = hepatitis B surface antigen, lnSII = Log-Transformed Systemic Immune-Inflammation Index, LYM = lymphocyte count, MO = monocyte count, Na = serum sodium, NEU = neutrophil count, PIR = poverty income ratio, PLT = platelet count, Scr = serum creatinine, TBil = total bilirubin.

### 3.2. The association between SII and all-cause mortality

During a mean follow-up of 105.8 ± 65.2 months, 682 mortality events (20.5%) occurred. Notably, the mortality rate in the highest SII quartile (Q4) was 24.9%, compared to 13.9% in the lowest quartile (Q1), underscoring the elevated risk associated with high SII. Unadjusted models demonstrated that lnSII significantly increased the risk of all-cause mortality (hazard ratio [HR] = 1.54, 95% CI: 1.22–1.94), as detailed in Table 2. After sequential multivariable adjustment, each 1-unit increment in lnSII maintained a persistent yet progressively attenuated association with elevated mortality risk. Specifically, demographic-adjusted analyses (Model 1) showed a 40% increased risk (HR = 1.40, 95% CI: 1.14–1.71), which adjusted to 38% (HR = 1.38, 95% CI: 1.10–1.73) with clinical factor incorporation (Model 2), and further attenuated to 35% (HR = 1.35, 95% CI: 1.09–1.68) under full covariate adjustment (Model 3). Compared to the lowest lnSII quartile (Q1), participants in the highest quartile (Q4) exhibited a significantly increased risk of all-cause mortality across all models. Hazard ratios were 1.57 (95% CI: 1.18–2.08; *P* < .001) for Model 1, 1.59 (1.17–2.16; *P* < .001) for Model 2, and 1.53 (1.13–2.08; *P* = .001) for Model 3. Full univariate Cox regression results are detailed in Table S1, Supplemental Digital Content, https://links.lww.com/MD/Q825.

**Table 2:** Hazard ratios for the association between quartiles of lnSII and all-cause mortality among US adults with HBV infection, NHANES 1999 to 2018.

	Q1	Q2	Q3	Q4	*P* for trend	lnSII
Unadjusted	1	0.89 (0.64–1.23)	1.20 (0.89–1.62)	1.64 (1.23–2.20)	<.001	1.54 (1.22–1.94)
Model 1	1	0.93 (0.68–1.27)	1.18 (0.89–1.57)	1.57 (1.18–2.08)	<.001	1.40 (1.14–1.71)
Model 2	1	0.94 (0.68–1.31)	1.22 (0.91–1.65)	1.59 (1.17–2.16)	<.001	1.38 (1.10–1.73)
Model 3	1	1.00 (0.72–1.39)	1.27 (0.94–1.70)	1.53 (1.13–2.08)	.001	1.35 (1.09–1.68)

Data are presented as HR (95% CI).

Model 1: adjusted for age, gender, education level, ethnicity, PIR.

Model 2: adjusted for age, gender, education level, ethnicity, PIR, smoking, ALP, AST, GGT, albumin, SCR, FIB-4.

Model 3: adjusted for age, gender, education level, ethnicity, PIR, smoking, ALP, AST, GGT, albumin, SCR, FIB-4, BMI, diabetes, HBP, cardiovascular, HBsAg.

ALP = alkaline phosphatase, AST = aspartate aminotransferase, BMI = body mass index, CI = confidence interval, FIB-4 = Fibrosis-4 Index, GGT = gamma-glutamyl transferase, HBsAg = hepatitis B surface antigen, HBV = hepatitis B virus, HR = hazard ratio, lnSII = Log-Transformed Systemic Immune-Inflammation Index, NHANES = National Health and Nutrition Examination Survey, PIR = poverty income ratio.

**Figure 2.**
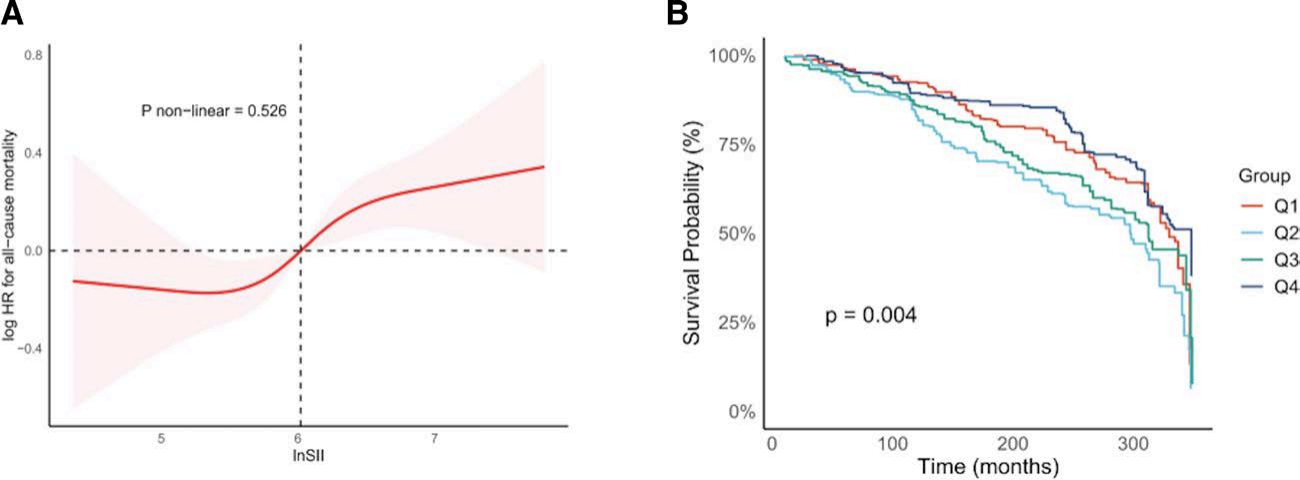
Dose–response association and survival analysis between SII and all-cause mortality. (A) Restricted cubic spline plot of lnSII and all-cause mortality, derived from a multivariable-adjusted Cox model. (B) Kaplan–Meier survival curves for participants stratified by quartiles of lnSII (Q1–Q4); survival distributions were compared using the log-rank test. lnSII = Log-Transformed Systemic Immune-Inflammation Index; SII= Systemic Immune-Inflammation Index.

To examine the dose–response relationship between lnSII and all-cause mortality, a restricted cubic spline regression was used to assess potential nonlinearity. Figure 2A demonstrates a nonsignificant nonlinear association after full covariate adjustment (P nonlinear 0.53), supporting a linear model characterization of this relationship (HR = 1.27, 95% CI: 1.11–1.46; *P* < .001; Table S2, Supplemental Digital Content, https://links.lww.com/MD/Q825). Concurrently, Kaplan–Meier curves in Figure 2B revealed significantly diminished survival probabilities and elevated mortality risk in the highest lnSII quartile (Q4) versus other strata (*P* = .004).

### 3.3. Superior prognostic utility of lnSII for all-cause mortality

**Figure 3.**
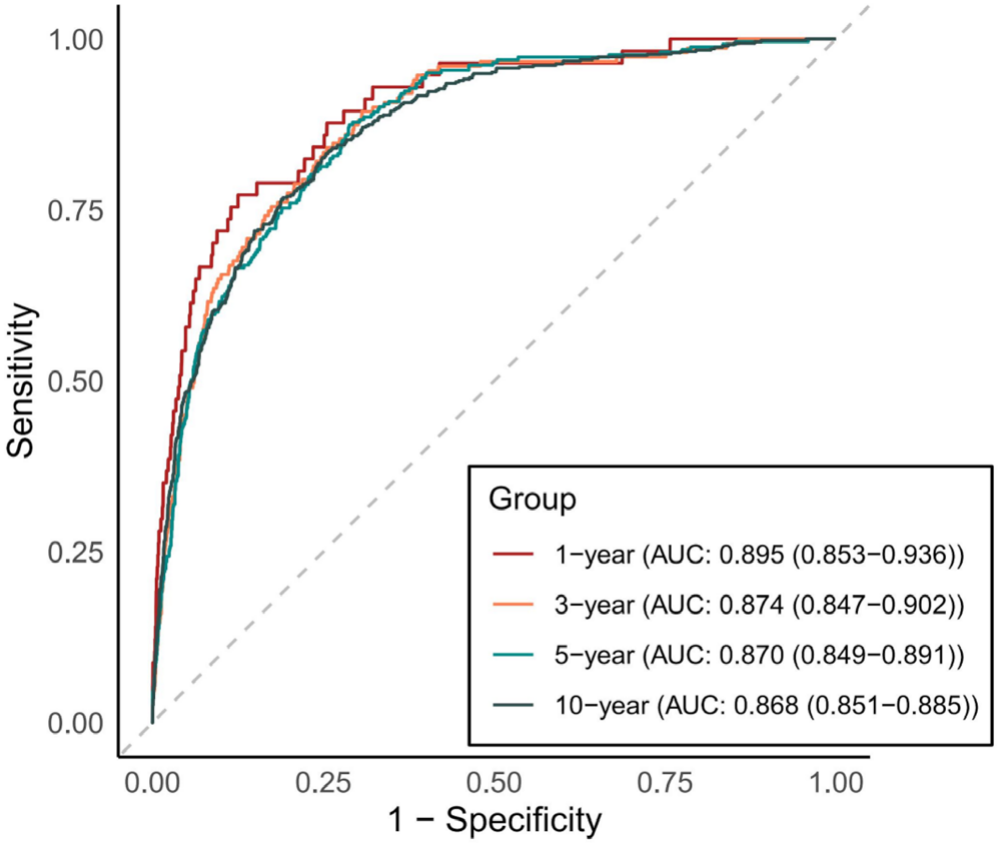
Time-dependent receiver operating characteristic curves for lnSII in predicting all-cause mortality. Curves were generated using the timeROC package in R, evaluating the discriminatory accuracy of the log-transformed Systemic Immune-Inflammation Index (lnSII) at 1, 3, 5, and 10 years. AUC = area under the curve; CI = confidence interval; lnSII = Log-Transformed Systemic Immune-Inflammation Index; ROC = receiver operating characteristic.

Figure 3 displays time-dependent receiver operating characteristic curves for lnSII in predicting all-cause mortality among HBV-infected individuals. The area under the curve (AUC) demonstrated sustained high discriminatory accuracy: 0.895 (95% CI: 0.853–0.936) at 1 year, 0.874 (0.847–0.902) at 3 years, 0.870 (0.849–0.891) at 5 years, and 0.868 (0.851–0.885) at 10 years.

### 3.4. Stratified subgroup analyses

**Figure 4.**
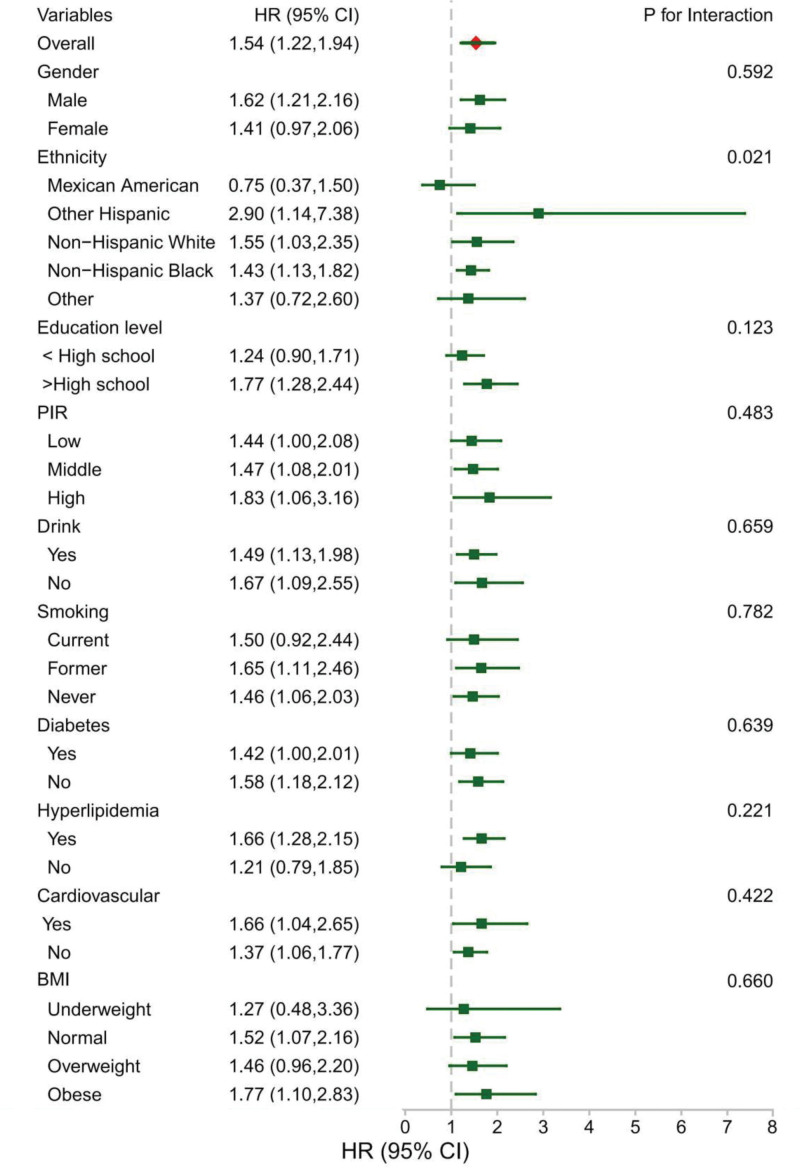
Subgroup analysis of the association between lnSII and all-cause mortality. Forest plot showing hazard ratios (HRs) per 1-unit increase in lnSII across subgroups. Estimates were obtained from Cox proportional hazards models within each stratum, and *P* values for interaction were calculated. HR = hazard ratio; lnSII = Log-Transformed Systemic Immune-Inflammation Index.

As shown in Figure 4, subgroup analyses demonstrated a consistent association between lnSII and all-cause mortality across most demographic and clinical strata. No significant interactions were observed for sex, household income, alcohol consumption, smoking status, diabetes, hypertension, cardiovascular disease, or BMI (all *P* > .05). Notably, considerable effect modification by race/ethnicity was detected (*P* < .05), with Hispanic individuals exhibiting the highest mortality risk elevation (HR = 2.90, 95% CI: 1.14–7.38). Table S3, Supplemental Digital Content, https://links.lww.com/MD/Q825 demonstrates concordance between the lnSII–mortality association and overall population estimates across all FIB-4 subgroups. Notably, in the low hepatic fibrosis stratum (FIB-4 < 1.3), each 1-unit increase in lnSII conferred a 201% elevated mortality risk (HR = 3.01, 95% CI: 2.07–4.38). RCS analysis in Figure S1, Supplemental Digital Content, https://links.lww.com/MD/Q826 further confirmed a significant U-shaped association between lnSII and mortality risk in the low hepatic fibrosis stratum (FIB-4 < 1.30; *P* nonlinear = .006), with the optimal prognostic window centered at lnSII 6.0 (SII ≈ 403).

### 3.5. Sensitivity assessments

Sensitivity analyses excluding deaths occurring within the initial 2 years yielded consistent findings (Table S4, Supplemental Digital Content, https://links.lww.com/MD/Q825). In the fully adjusted Cox regression model using the lowest quartile (Q1) as reference, individuals in the highest SII quartile (Q4) maintained significantly elevated mortality risk (HR = 1.59, 95% CI: 1.13–2.24; *P* = .004). The proportion of missing data and diagnostics of multiple imputation are detailed in Table S5, Supplemental Digital Content, https://links.lww.com/MD/Q825 and Figure S2, Supplemental Digital Content, https://links.lww.com/MD/Q826. Across all 10 imputed datasets (Figure S3, Supplemental Digital Content, https://links.lww.com/MD/Q826), SII consistently demonstrated directionally concordant associations with all-cause mortality, confirming the robustness of our conclusions against potential missing data bias. To quantify the potential impact of unmeasured confounding, we conducted *E*-value sensitivity analyses. As shown in Figure S4, Supplemental Digital Content, https://links.lww.com/MD/Q826, the adjusted hazard ratio (HR) for lnSII-associated all-cause mortality was 1.35 (95% CI: 1.09–1.68 as reported), with an *E*-value of 1.76.

## 4. Discussion

This large-scale, nationally representative cohort study comprehensively evaluated the prognostic utility of the Systemic SII for all-cause mortality in individuals infected with HBV. We demonstrated that elevated SII independently predicted significantly increased mortality risk, with this association remaining robust after rigorous multivariable adjustment. SII exhibited consistent predictive capacity across sensitivity analyses and stratified subgroups, confirming its reliability for risk stratification in clinical practice. Furthermore, our findings highlight the clinical utility of SII for personalized risk assessment and precision medicine in HBV infection. As a composite biomarker derived from routine blood tests, SII is cost-effective and easily obtainable, supporting its broad implementation for risk stratification across diverse healthcare settings. Its consistently high discriminatory accuracy for predicting mortality over both short- and long-term intervals (with AUCs exceeding 0.86 at 1, 3, 5, and 10 years) enables dynamic risk evaluation throughout the disease continuum. Subgroup analyses further identified clinically meaningful variations, including heightened risk among Hispanic individuals and a U-shaped relationship in patients with minimal hepatic fibrosis (FIB-4 < 1.30). These observations indicate that SII can discern distinct risk profiles, thereby informing ethnicity-specific and stage-adjusted monitoring and intervention strategies. Integrating SII into established prognostic frameworks may thus improve early detection of high-risk individuals, guide personalized treatment approaches (such as intensified anti-inflammatory or antiviral therapy) and optimize follow-up resource allocation. To our knowledge, this represents the 1st investigation leveraging NHANES cohort data to establish SII as a novel predictor of all-cause mortality in the HBV-infected population.

The liver, functioning as a pivotal immunological organ, performs multifaceted roles including antigen recognition, immune tolerance induction, and inflammatory regulation.^[27]^ In chronic HBV infection, persistent immune response dysregulation constitutes the central pathogenic mechanism driving hepatic injury and disease progression.^[28,29]^ SII serves as a composite biomarker that precisely quantifies this dysregulated state. Neutrophils exacerbate hepatocellular injury and fibrogenesis within the hepatic inflammatory microenvironment through reactive oxygen species generation, degranulation, cytokine/chemokine production, and neutrophil extracellular traps formation.^[30]^ Platelets not only mediate coagulation but also amplify hepatic inflammation and fibrosis by secreting chemokines that recruit inflammatory cells.^[31]^ In parallel, reduced lymphocyte counts reflect exhaustion of virus-specific immunity, compromising viral clearance and perpetuating chronic liver injury.^[32]^ Consequently, the concurrent pro-inflammatory and immunosuppressive states captured by elevated SII collectively constitute the biological basis for adverse clinical outcomes in HBV-infected individuals. Paradoxically, platelets liberate multiple growth factors, including vascular endothelial growth factor and platelet-derived growth factor that execute critical roles in hepatic repair following injury.^[33,34]^ Furthermore, regulatory T cells play a dual role in HBV pathogenesis. Their frequency and function are often enhanced in chronic infection.^[35]^ Through cell-contact-dependent mechanisms and secretion of immunosuppressive cytokines (IL-10, TGF-β), they suppress hyperactivation of effector T cells to prevent immunopathology-mediated hepatocyte damage. However, this same immunosuppressive activity can also inhibit effective viral clearance, thereby facilitating viral persistence.

Accumulating evidence has established that elevated SII correlates strongly with adverse clinical outcomes in chronic liver disease. For instance, a prospective cohort study by Ma et al involving 288 patients with chronic hepatitis C demonstrated SII as an independent predictor of liver fibrosis progression.^[36]^ Similarly, a multicenter investigation revealed that heightened SII portends poorer survival outcomes in HCC patients, further validating its prognostic utility.^[37]^ Current biomarkers for systemic inflammation include C-reactive protein, erythrocyte sedimentation rate, neutrophil-to-lymphocyte ratio, and platelet-to-lymphocyte ratio. In HCC research (a major sequela of chronic HBV) SII demonstrates superior prognostic capability compared to conventional indices. One validation study reported that SII provided higher discriminatory accuracy for overall survival (AUC 0.66) than established ratios and remained an independent predictor of mortality (HR 2.10, *P* = .017) in multivariable analyses incorporating both neutrophil-to-lymphocyte ratio and platelet-to-lymphocyte ratio.^[38]^ These findings indicate that SII more comprehensively captures the interplay between pro-inflammatory and immune states. Moreover, persistent inflammatory activation not only exacerbates hepatic damage but also disrupts systemic immune homeostasis, elevating risks of multi-system complications such as infections and cardiovascular events, thereby increasing all-cause mortality.^[39,40]^

A systematic review of > 9000 patients confirmed stepwise progression of hepatic fibrosis during follow-up in chronic HBV infection.^[41]^ Research by Mayorca-Guiliani et al and Li et al revealed U-shaped modulation of key extracellular matrix remodeling mediators, including matrix metalloproteinase-2 and urokinase plasminogen activator, concurrently with the NRF-2/HO-1 signaling pathway in early-stage fibrosis, reflecting a dynamic imbalance between reparative and injurious processes.^[42,43]^ Complementary studies established that the inflammatory mediator TNF-α bidirectionally regulates fibrogenesis through a threshold effect where low concentrations promote tissue regeneration while elevated levels drive an NF-κB-mediated self-perpetuating cycle.^[44,45]^ Serum biomarkers such as type IV collagen (>3 × upper limit of normal) and hyaluronic acid demarcate early-stage hepatic fibrosis through threshold effects.^[46]^ This study identified a specific U-shaped nonlinear association between SII and early fibrotic progression, establishing it as a dynamic diagnostic threshold that enables stage-specific therapeutic interventions.

Mechanistic evidence reveals that functionally characterized polymorphisms in metabolizing-enzyme genes, enriched in Hispanic populations, potentiate oxidative stress-mediated damage, predisposing to exacerbated immune dysregulation in chronic HBV infection.^[47,48]^ Consequently, neglecting ethnicity-specific pathophysiological crosstalk will perpetuate health inequities, mandating large-scale multiethnic cohorts to implement equitable precision hepatology frameworks. Furthermore, we observed elevated SII levels among non-Hispanic Black individuals. Although the underlying mechanisms are likely multifactorial and cannot be definitively elucidated here, several plausible explanations warrant consideration. First, socioeconomic disparities (including limited healthcare access, delayed diagnosis, and suboptimal antiviral therapy adherence) are more common in non-Hispanic Black communities in the U.S.^[49]^ These factors may promote persistent viral replication and chronic hepatic inflammation, thereby increasing SII. Second, a higher prevalence of pro-inflammatory comorbidities, such as hypertension, obesity, and diabetes, in this population may synergistically amplify systemic inflammation in the context of HBV infection.^[50]^ Finally, immunogenetic differences may contribute. Growing evidence indicates that populations of diverse ancestry vary in their baseline immune homeostasis and carry polymorphisms in genes involved in innate immunity and neutrophil regulation.^[51,52]^ Such genetic backgrounds could predispose non-Hispanic Black individuals to heightened inflammatory responses during chronic HBV infection. While these hypotheses remain speculative and require validation in future studies, they underscore the complex interplay among social determinants of health, comorbid disease, and host biology in shaping inflammatory milieus and clinical outcomes in HBV infection.

Leveraging a large-scale nationally representative sample, this study employed comprehensive adjustment for demographic, clinical, laboratory, and lifestyle covariates, substantially reducing potential confounding. The robustness of the findings was further supported by sensitivity analyses, including *E*-value assessment, which indicated that an unmeasured confounder would need to be associated with both lnSII and mortality by a risk ratio of ≥ 1.76 to fully explain the observed association (HR = 1.35). Given the extensive adjustment for known major confounders, such a strong residual confounder is unlikely, strengthening causal inference. Additionally, subgroup analyses consistently corroborated the associations across diverse populations and clinical profiles.

This study has some inherent limitations. First, our study cohort comprised a limited number of HBV-infected participants. The prevalence of HBV infection in our source data (∼3.3%) was somewhat lower than recent national estimates for anti-HBc seropositivity (∼4.3%). This discrepancy may reflect the underrepresentation of institutionalized populations, where HBV prevalence is likely higher. Consequently, the generalizability of our findings to such groups may be limited. Second, the analysis of cause-specific mortality was constrained by the public-use linked mortality files. As per the National Center for Health Statistics documentation, the underlying cause of death is available only as a consolidated variable (UCOD_LEADING) that groups liver-related deaths into a residual “all other causes” category, precluding a direct calculation of the HBV-attributable mortality fraction. However, established literature indicates that liver-related complications account for approximately 15% to 30% of all deaths in chronic HBV infection,^[53]^ which supports the biological plausibility that a substantial portion of the SII-associated mortality risk is mediated through hepatic sequelae. Third, the lack of a defined minimum abstinence period for former smokers introduces heterogeneity, as health risks differ between recent and long-term quitters. Fourth, SII values derived from single-timepoint blood measurements fail to capture the temporal dynamics of systemic inflammation. Finally, the observational design precludes definitive establishment of causal relationships between SII and mortality. Therefore, prospective cohort studies incorporating serial biomarker assessments and standardized smoking behavior definitions are warranted to clarify the longitudinal association and prognostic utility of SII in HBV-infected populations.

## 5. Conclusions

Using a nationally representative sample of 3332 U.S. adults with HBV infection from NHANES, we demonstrated that SII strongly predicts all‑cause mortality. Individuals in the highest SII quartile (Q4) had a significantly elevated risk of death, which remained robust after adjustment for demographic and clinical confounders. SII also showed high predictive accuracy for both short‑ and long‑term mortality, with time‑dependent AUCs consistently above 0.86. These results support SII as a novel, accessible biomarker for improving risk stratification and prognosis in HBV infection. Prospective studies that track serial changes in liver fibrosis along with SII trajectories are needed to confirm its utility in personalized patient management.

## Acknowledgments

The authors gratefully acknowledge the National Center for Health Statistics (NCHS) for sustained efforts in data collection, curation, and public dissemination across survey cycles. Special gratitude is extended to Professor Gang Wu for his invaluable guidance on study conceptualization, statistical design, and critical manuscript revisions, which proved instrumental to the successful completion of this research.

## Author contributions

**Conceptualization**: Min Chen, Jiameng Li, Gang Wu.

**Data curation**: Min Chen, Jiameng Li, Li Qiang.

**Formal analysis**: Jiameng Li.

**Funding acquisition**: Li Qiang, Gang Wu.

**Investigation**: Yu Tang.

**Methodology**: Min Chen, Yu Tang, Jiameng Li.

**Project administration**: Li Qiang, Gang Wu.

**Resources**: Li Qiang.

**Supervision**: Li Qiang, Gang Wu.

**Software**: Min Chen.

**Validation**: Yu Tang.

**Visualization**: Min Chen, Yu Tang, Jiameng Li.

**Writing – original draft**: Min Chen.

**Writing – review & editing**: Min Chen.

## Supplementary Material
