# Supplementary material for: Systemic immune-inflammation index as a predictor of all-cause mortality in patients with hepatitis B virus infection: A cross-sectional study based on NHANES 1999 to 2018
Source: Medicine (Baltimore). 2026 May 12;104(49):e46305. doi: 10.1097/MD.0000000000046305 (PMC12688859; doi:10.1097/MD.0000000000046305)
Supplement: Supplementary file 2 [file medi-104-e46305-s002.docx]

Supplemental Table 1. Univariable Cox Regression Analysis of Factors Associated with All-Cause Mortality Among US Adults with HBV Infection, NHANES 1999–2018.

| Variable | HR（95%CI） | P-value |
| --- | --- | --- |
| Age | 1.09 (1.08-1.09) | <0.001 |
| Gender |  |  |
| Male | Ref |  |
| Female | 0.78 (0.67-0.91) | 0.002 |
| Ethnicity |  |  |
| Mexican American | Ref |  |
| Other Hispanic | 0.54 (0.35-0.85) | 0.008 |
| Non-Hispanic White | 1.94 (1.44-2.60) | <0.001 |
| Non-Hispanic Black | 1.32 (0.99-1.76) | 0.061 |
| Other | 0.64 (0.45-0.90) | 0.01 |
| Education level |  |  |
| <High school | Ref |  |
| >High school | 0.59 (0.51-0.69) | <0.001 |
| PIR |  |  |
| Low | Ref |  |
| Middle | 1.08 (0.91-1.29) | 0.387 |
| High | 0.64 (0.51-0.80) | <0.001 |
| Smoking |  |  |
| Never | Ref |  |
| Current | 1.23 (1.01-1.50) | 0.036 |
| Former | 1.85 (1.55-2.19) | <0.001 |
| Drink |  |  |
| No | Ref |  |
| Yes | 1.06 (0.91-1.25) | 0.447 |
| BMI |  |  |
| Underweight | Ref |  |
| Normal | 0.48 (0.31-0.74) | 0.001 |
| Overweight | 0.55 (0.36-0.84) | 0.006 |
| Obese | 0.56 (0.37-0.87) | 0.009 |
| Diabetes |  |  |
| No | Ref |  |
| Yes | 2.27 (1.93-2.66) | <0.001 |
| Hyperlipidemia |  |  |
| No | Ref |  |
| Yes | 2.74 (2.32-3.22) | <0.001 |
| Cardiovascular |  |  |
| No | Ref |  |
| Yes | 3.05 (2.51-3.71) | <0.001 |
| HBsAg |  |  |
| Negative | Ref |  |
| Positive | 0.66 (0.46-0.94) | 0.023 |
| NEU | 1.11 (1.06-1.16) | <0.001 |
| LYM | 0.69 (0.61-0.78) | <0.001 |
| MO | 3.10 (2.49-3.85) | <0.001 |
| PLT | 1.00 (1.00-1.00) | <0.001 |
| ALT | 1.00 (1.00-1.00) | 0.579 |
| AST | 1.00 (1.00-1.00) | 0.001 |
| GGT | 1.00 (1.00-1.00) | <0.001 |
| ALP | 1.01 (1.01-1.01) | <0.001 |
| Albumin | 0.87 (0.85-0.89) | <0.001 |
| TBil | 1.00 (0.98-1.02) | 0.982 |
| SCR | 1.00 (1.00-1.00) | <0.001 |
| Na | 1.02 (0.99-1.06) | 0.156 |
| FIB-4 | 1.18 (1.16-1.20) | <0.001 |
| lnSII |  |  |
| Q1 | Ref |  |
| Q2 | 1.02 (0.80-1.30) | 0.875 |
| Q3 | 1.32 (1.06-1.65) | 0.015 |
| Q4 | 1.60 (1.29-1.98) | <0.001 |

*NEU, Neutrophil Count; LYM, Lymphocyte Count; MO, Monocyte Count; PLT, Platelet Count; ALT, Alanine Aminotransferase; AST, Aspartate Aminotransferase; GGT, Gamma-Glutamyl Transferase; ALP, Alkaline Phosphatase; TBil, Total Bilirubin; Scr, Serum Creatinine; Na, Serum Sodium; FIB-4, Fibrosis-4 Index; PIR, Poverty-Income Ratio; HBsAg, Hepatitis B Surface Antigen; lnSII, log-transformed Systemic Immune-Inflammation Index.

Supplemental Table 2. Adjusted Linear Hazard Ratio for All-Cause Mortality per Unit Increase in lnSII Among US Adults with HBV Infection, NHANES 1999–2018.

| Variable | HR (95% CI) | P-Value |
| --- | --- | --- |
| lnSII | 1.27（1.11，1.46） | <0.001 |

*Adjust for Age, Gender, Education level, Ethnicity, PIR, Smoking, ALP, AST, GGT, Albumin, SCR, FIB-4, BMI, Diabetes, HBP, Cardiovascular, HbsAg.

Supplemental Table 3. Sensitivity Analysis: Association Between lnSII and All-Cause Mortality, Stratified by FIB-4 Index, Among US Adults with HBV Infection, NHANES 1999–2018.

|  | <1.30 | 1.30-2.67 | >2.67 | P for Interaction |
| --- | --- | --- | --- | --- |
| All-cause Mortality | 3.01 (2.07,4.38) | 3.01 (2.07,4.38) | 3.01 (2.07,4.38) | 0.0673 |

*Data are expressed as HR (95%CI)

Supplemental Table 4. Sensitivity Analysis: Hazard Ratios for the Association Between lnSII and All-Cause Mortality After Excluding Early Deaths (First 2 Years) Among US Adults with HBV Infection, NHANES 1999–2018.

| Model | Q1 | Q2 | Q3 | Q4 | P for trend | lnSII |
| --- | --- | --- | --- | --- | --- | --- |
| Unadjusted | 1 (Reference) | 0.99 (0.70,1.41) | 1.26 (0.91,1.74) | 1.59 (1.15,2.19) | 0.001 | 1.36 (1.07,1.73) |
| Model1 | 1 (Reference) | 1.08 (0.77,1.51) | 1.26 (0.92,1.72) | 1.56 (1.14,2.14) | 0.002 | 1.27 (1.03,1.56) |
| Model2 | 1 (Reference) | 1.08 (0.76,1.54) | 1.31 (0.95,1.81) | 1.61 (1.15,2.26) | 0.002 | 1.27 (1.01,1.60) |
| Model3 | 1 (Reference) | 1.15 (0.80,1.65) | 1.35 (0.98,1.87) | 1.59 (1.13,2.24) | 0.004 | 1.27 (1.01,1.60) |

*Data are presented as HR (95%CI). Model 1: Adjust for Age, Gender, Education level, Ethnicity, PIR. Model 2: Adjust for Age, Gender, Education level, Ethnicity, PIR, Smoking, ALP, AST, GGT, Albumin, SCR, FIB-4. Model 3: Adjust for Age, Gender, Education level, Ethnicity, PIR, Smoking, ALP, AST, GGT, Albumin, SCR, FIB-4, BMI, Diabetes, HBP, Cardiovascular, HbsAg.

Supplemental Table 5. Sensitivity Analysis: Number and Proportion of Missing Data for Key Covariates in the Study of lnSII and Mortality Among US Adults with HBV Infection, NHANES 1999–2018.

| Variable | Missing | Missing proportion |
| --- | --- | --- |
| Drinking | 495 | 14.86% |
| PIR | 364 | 10.92% |
| BMI | 76 | 2.28% |
| Education level | 48 | 1.44% |
| Smoking | 40 | 1.20% |
