# Supplementary figures and images for: Systemic immune-inflammation index as a predictor of all-cause mortality in patients with hepatitis B virus infection: A cross-sectional study based on NHANES 1999 to 2018
Source: Medicine (Baltimore). 2026 May 12;104(49):e46305. doi: 10.1097/MD.0000000000046305 (PMC12688859; doi:10.1097/MD.0000000000046305)

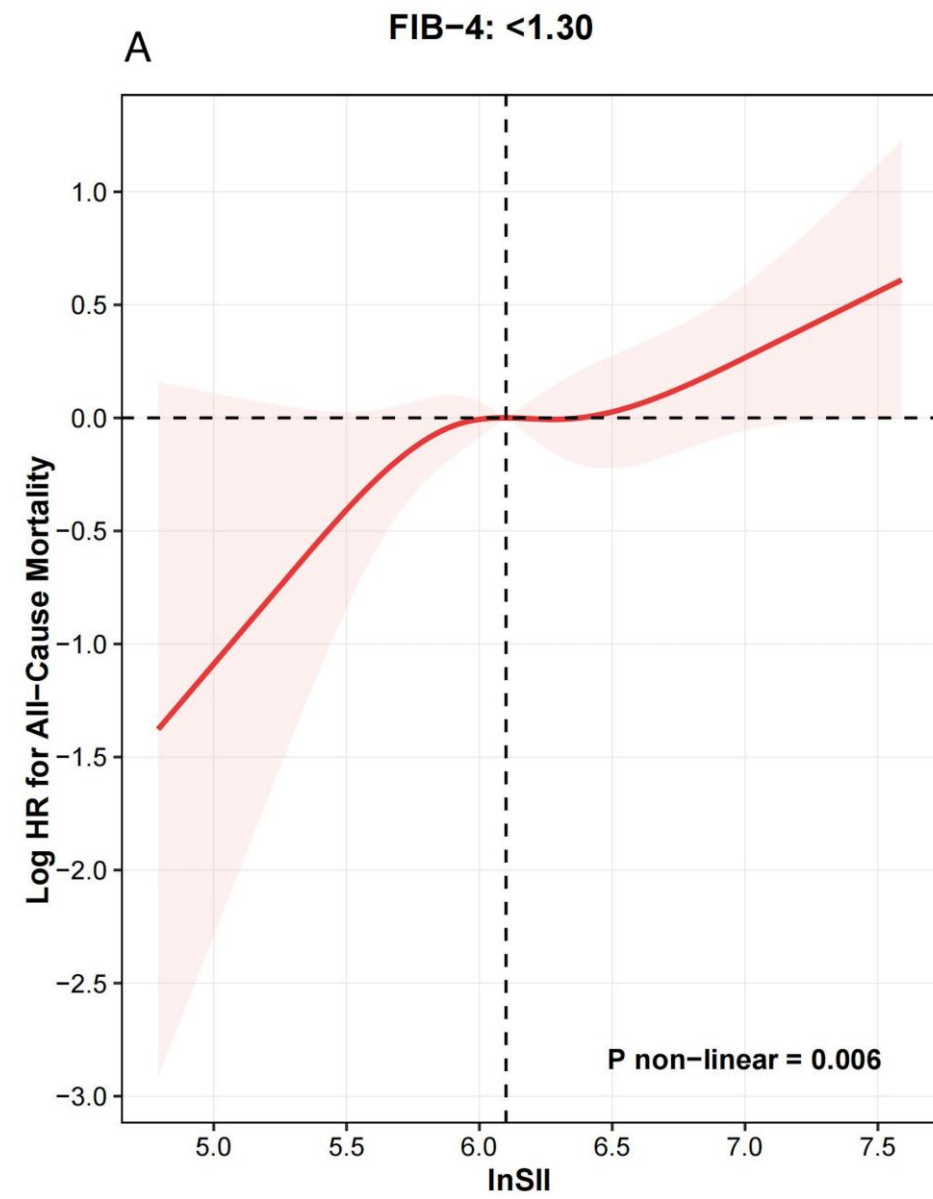

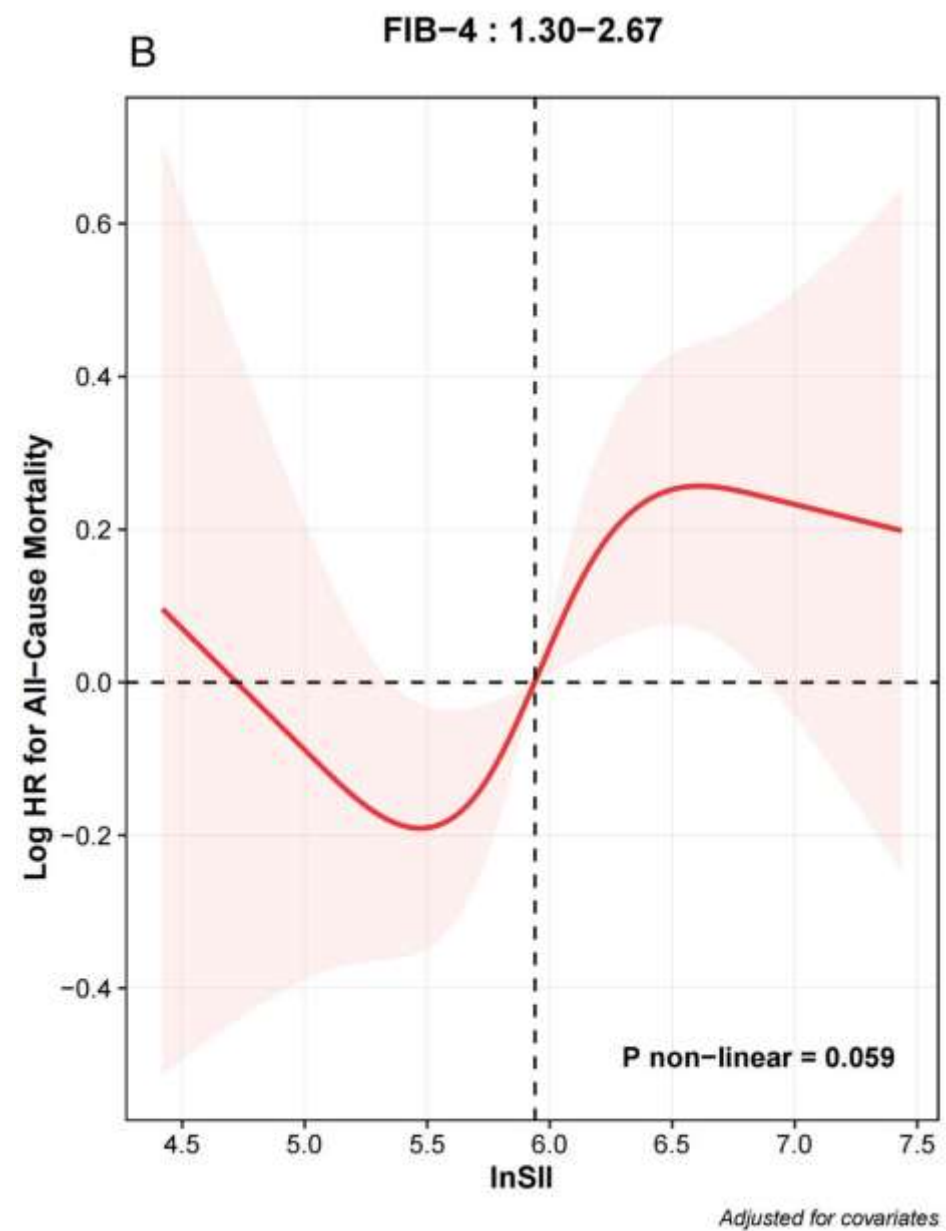

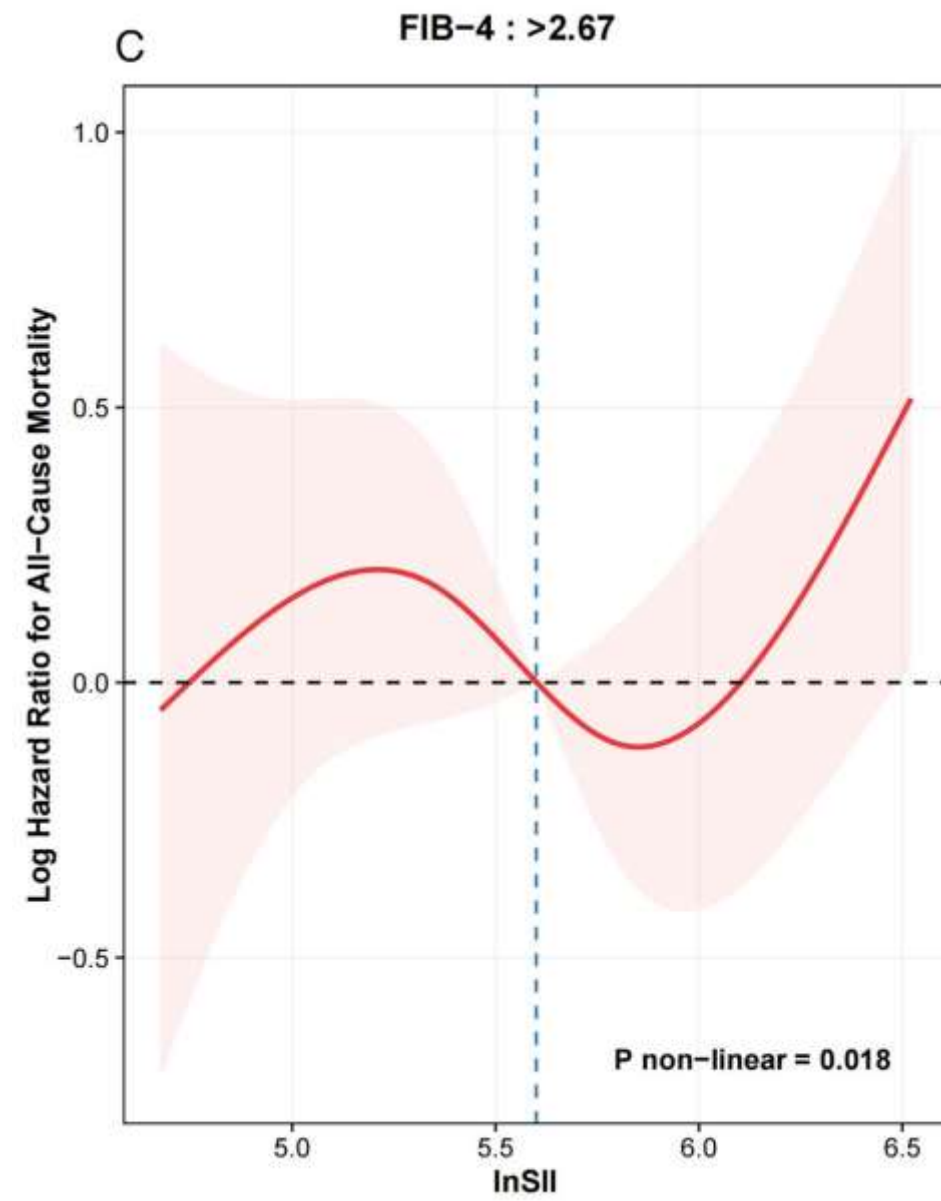

A

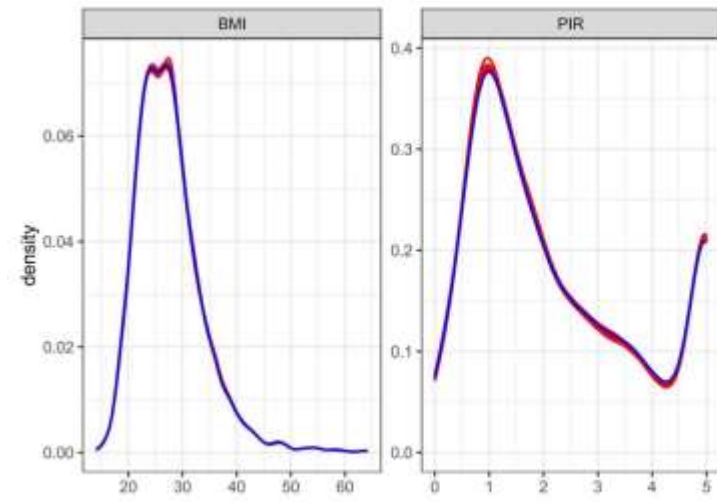

B

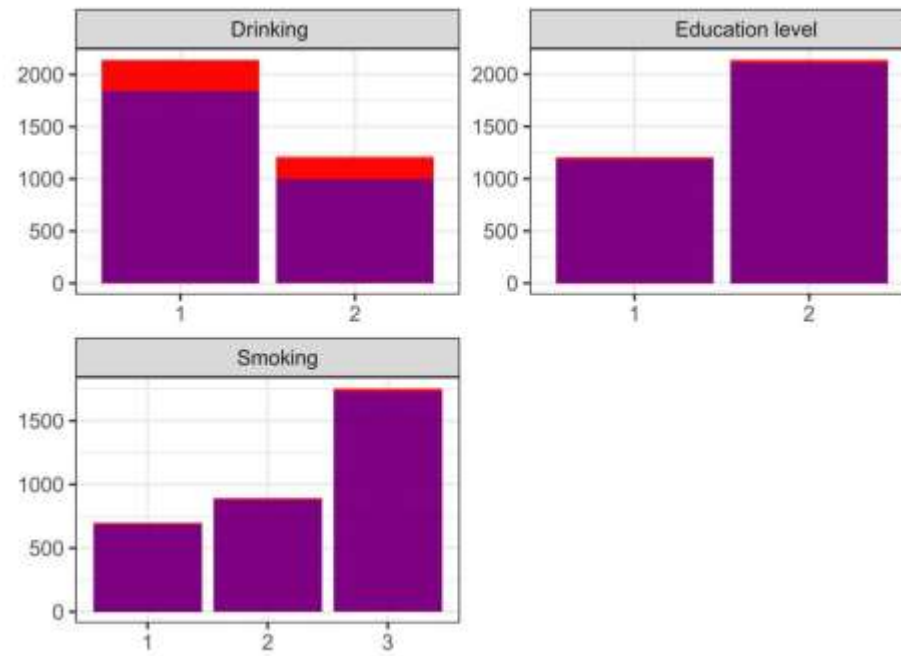

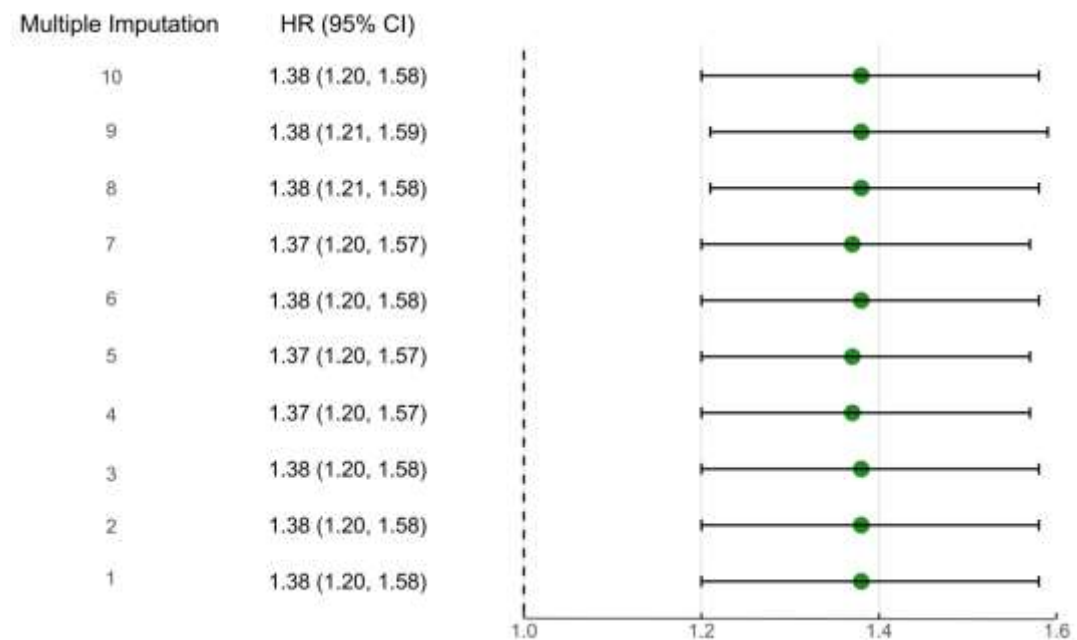

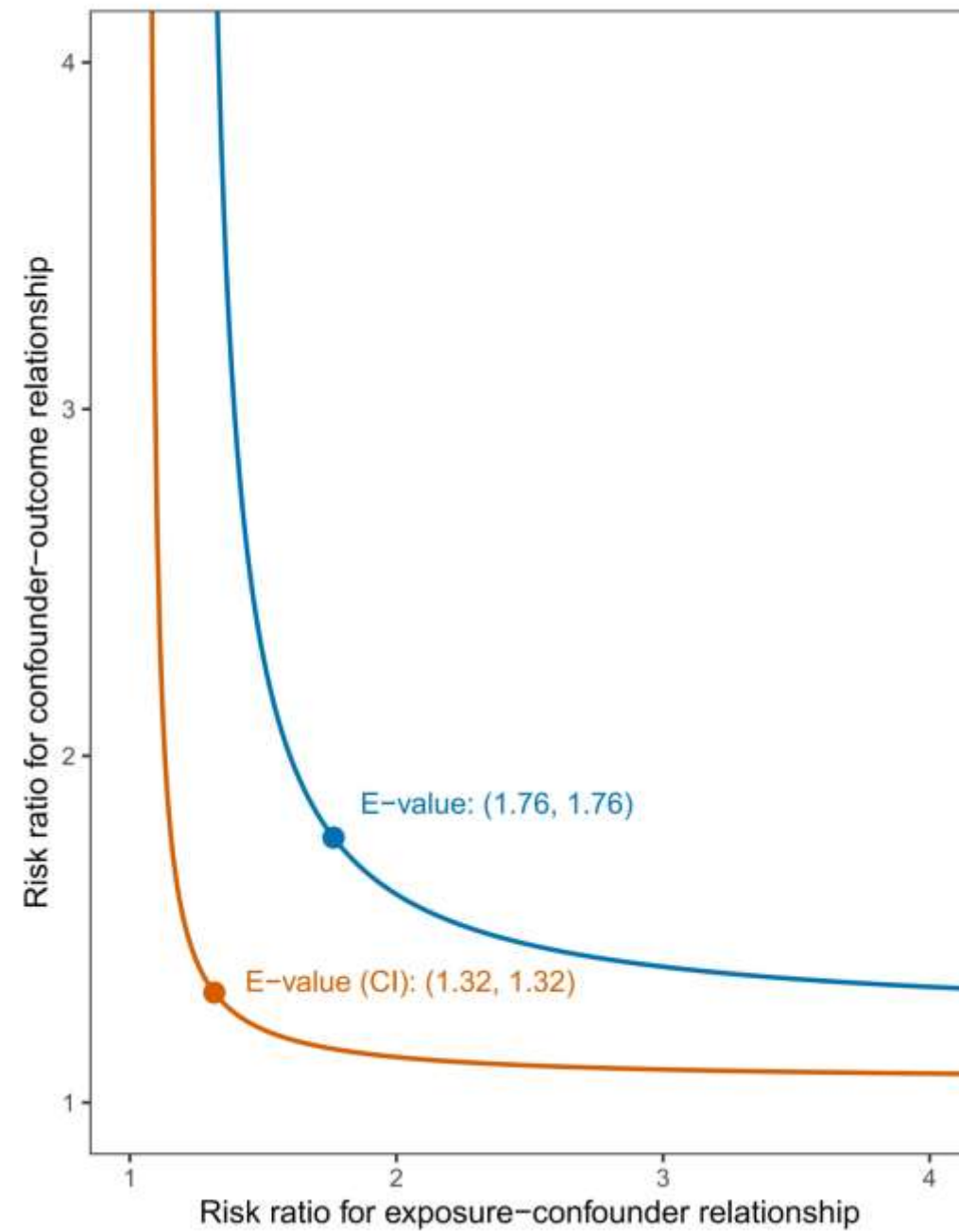

Supplement: Supplementary file 3 [file medi-104-e46305-s003.pdf]
